# Right Inferior Frontal Activation During Alcohol-Specific Inhibition Increases With Craving and Predicts Drinking Outcome in Alcohol Use Disorder

**DOI:** 10.3389/fpsyt.2022.909992

**Published:** 2022-07-01

**Authors:** Matthias Grieder, Leila M. Soravia, Raphaela M. Tschuemperlin, Hallie M. Batschelet, Andrea Federspiel, Simon Schwab, Yosuke Morishima, Franz Moggi, Maria Stein

**Affiliations:** ^1^Translational Research Center, University Hospital of Psychiatry and Psychotherapy, University of Bern, Bern, Switzerland; ^2^Clinic Suedhang, Kirchlindach, Switzerland; ^3^Department of Clinical Psychology and Psychotherapy, Institute of Psychology, University of Bern, Bern, Switzerland

**Keywords:** alcohol use disorder (AUD), craving, drinking outcome, fMRI, Go-NoGo, inferior frontal gyrus (IFG), inhibition, inhibition training

## Abstract

Alcohol use disorder (AUD) is characterized by enhanced cue-reactivity and the opposing control processes being insufficient. The ability to inhibit reactions to alcohol-related cues, alcohol-specific inhibition, is thus crucial to AUD; and trainings strengthening this ability might increase treatment outcome. The present study investigated whether neurophysiological correlates of alcohol-specific inhibition (I) vary with craving, (II) predict drinking outcome in AUD and (III) are modulated by alcohol-specific inhibition training. A total of 45 recently abstinent patients with AUD and 25 controls participated in this study. All participants underwent functional magnetic resonance imaging (fMRI) during a Go-NoGo task with alcohol-related as well as neutral conditions. Patients with AUD additionally participated in a double-blind RCT, where they were randomized to either an alcohol-specific inhibition training or an active control condition (non-specific inhibition training). After the training, patients participated in a second fMRI measurement where the Go-NoGo task was repeated. Percentage of days abstinent was assessed as drinking outcome 3 months after discharge from residential treatment. Whole brain analyses indicated that in the right inferior frontal gyrus (rIFG), activation related to alcohol-specific inhibition varied with craving and predicted drinking outcome at 3-months follow-up. This neurophysiological correlate of alcohol-specific inhibition was however not modulated by the training version. Our results suggest that enhanced rIFG activation during alcohol-specific (compared to neutral) inhibition (I) is needed to inhibit responses when craving is high and (II) fosters sustained abstinence in patients with AUD. As alcohol-specific rIFG activation was not affected by the training, future research might investigate whether potential training effects on neurophysiology are better detectable with other methodological approaches.

## Introduction

Alcohol use disorder (AUD) is a leading cause for societal and individual burden of disease (1, 2) and treatment still needs to be improved (3). Central to the disorder is the fact that patients with AUD repeatedly fail to inhibit or control their drinking and continue drug use despite negative consequences. Establishing an ability to resist drinking urges and inhibit drinking behavior is thus of major importance for AUD treatment. While multiple brain networks are implicated in AUD (4–6), two processes seem crucial when it comes to inhibition in an alcohol-related context. Neuroscientific models postulate that, on the one hand, cue reactivity and subjective craving in response to alcohol-related stimuli is too strong; On the other hand, control processes are too weak to inhibit resulting drinking urges (4, 6, 7). These models, as well as clinical experience, thus suggest that inhibitory control is especially relevant in the context of the opposing appetitive processes, making alcohol-specific inhibition particularly important for AUD.

On a behavioral level, patients with AUD displayed inhibitory deficits (8–10) when their performance on inhibitory control tasks such as the Go-NoGo (GNG) task and the stop signal task was compared against healthy control groups. These deficits were reported to be pronounced in an alcohol-related compared to a neutral context (11–14). On a neurophysiological level, response inhibition is typically supported by a right lateralized fronto-striatal-parietal network (15, 16), which seems to be dysregulated in AUD (4, 5, 17, 18). The response inhibition network includes dorsolateral and ventrolateral prefrontal cortices, medial frontal regions (pre-SMA and ACC), thalamus, dorsal striatum, and the inferior parietal lobe (18). While most of these areas are involved in a broad variety of cognitive control tasks, the right ventrolateral prefrontal cortex (IFG, particularly BA 44/45) seems to be crucial for response inhibition (19–21) and specifically activated by response inhibition tasks (22, 23).

Dysregulations in the inhibitory control network in AUD and other addictive disorders have been repeatedly observed (5, 17, 18) in prefrontal, parietal and cingulate regions as well as in basal ganglia (24–30). Inconsistencies regarding the direction of this dysregulation (hypo- or hyperactivation) have been attributed to differences in task design and analytic strategy (17), to the extent of performance deficits (5, 31) and to variations in the stimulus material used (4). When assessed with GNG or stop-signal-tasks, hyperactivation during inhibitory control has rather been reported when addiction-related stimuli are used (24, 29, 31) and/or no behavioral performance differences are observed (25, 26, 29, 31–34), while hypoactivation was rather observed in studies using neutral stimuli (30, 35–38) and/or also reporting performance deficits (35, 36). This might be indicative of a general impairment of the inhibitory system, which is hypo-activated and less responsive unless confronted with addiction-related cues and/or charged with functional compensation in order to achieve near-normal task performance (4, 5, 26, 31).

Following the logic that response inhibition is especially crucial in the context of alcohol-related cues, which may trigger strong appetitive processes (4, 39–41), some studies have investigated alcohol-specific inhibition. When brain activation during inhibition in an alcohol-related context is directly compared to neutral inhibition, AUD seems to be characterized by increased neural activation during alcohol-related inhibition (29, 31). Furthermore, this alcohol-specific inhibitory activation was observed to increase with craving (31, 42, 43), suggesting that alcohol-specific inhibition is especially effortful in subjects experiencing high craving. Moreover, two studies reported that electrophysiological correlates of alcohol-specific inhibition discriminated between patients which relapsed and those who remained abstinent in the following 3-month period (43, 44), hinting at the potential clinical relevance of this specific subtype of inhibition. A potential linkage between drinking outcomes and the functional neuroanatomy of alcohol-specific inhibition, as assessed with fMRI, has not yet been investigated.

Taken together, neuroscientific and experimental research in AUD suggests that inhibition is crucial, and probably also particularly difficult, when it must be exerted in an alcohol-related context which provokes craving.

Following a translational approach, such research led to the development of an alcohol-specific inhibition training (Alc-IT), which was designed to improve patients' inhibitory control over their responses to alcohol-related stimuli (45, 46). Alc-IT led to mixed, but nonetheless promising, results in a series of non-clinical proof-of-concept studies (3). Recently, a first clinical RCT tested its effects as an add-on to treatment as usual (47) and reported positive effects for an improved variant of Alc-IT. This improved Alc-IT operates like a modified GNG task with a high Go/NoGo-ratio and selectively pairs alcohol-related pictures with NoGo cues, while neutral pictures are paired with Go cues. Thus, it establishes a prepotent response tendency, which then must be inhibited in the context of alcohol-related stimuli. The precise working mechanism of improved Alc-IT is still being debated (3), one proposition holds that it works *via* the improvement of alcohol-specific inhibitory capacities. Such a working mechanism would potentially also induce changes in the neurophysiological correlates of alcohol-specific inhibition.

The presented study thus aims to (I) replicate earlier reports indicating that neuronal activation related to alcohol-specific inhibition increases with craving; (II) test whether neuronal activation related to alcohol-specific inhibition is related to drinking outcomes and (III) investigate whether the neurophysiological signature of alcohol-specific inhibition can be altered through alcohol-specific inhibition training.

## Materials and Methods

### Procedure

In the context of a randomized-controlled, double-blind, clinical trial investigating the effects of alcohol-specific inhibition training on drinking outcomes (47), the present paper reports on an additional sub-study, which investigated the neuronal correlates of the improved version of this inhibition training. For this sub-study, 49 patients were recruited to participate in a longitudinal multimodal MRI-study. Only fMRI data were included in the present analyses and are described in detail. All patients were recruited at the beginning of their residential treatment program. During the second treatment week, a baseline measurement comprised questionnaires, diagnostics, and a Timeline-Follow-Back interview (TLFB). About one to two weeks later, a pre-training assessment comprised questionnaires and a multimodal MRI measurement, which also comprised fMRI measurement during participation in a GNG task. An independent investigator then randomly assigned the patients to one of two computerized training interventions using block randomization with variable block sizes [stratified according to gender and age (age groups: 18–25, 26–35, 36–45, 46–55, and 56–60)] following a randomization list generated with MATLAB (version 2017a, Mathworks, Natick, USA). The list was stored in a locked place; thus keeping participants, care providers, investigators and members of the study team blind to the allocation. During treatment weeks 4 and 5, all patients participated in six short (~10–15 min) training sessions of their allocated condition (improved Alc-IT, or control training). The patients' average reaction times and error rates were communicated after each training session to maintain motivation. Between 1 and 4 days after the last training session, a post-training assessment comprised the same measures as the pre-training assessment, including the fMRI session. Patients were then to complete their residential treatment (~8–12 weeks in total). Upon discharge from residential treatment, a questionnaire battery was administered. Three months after discharge from treatment, all patients were contacted by mail and by telephone and primary outcome variables for the 3-month follow-up were assessed in a short telephone interview, a TLFB interview, and a questionnaire battery. See Tschuemperlin et al. (47) for detailed study protocol of the main study.

### Participants

**Figure 1:**
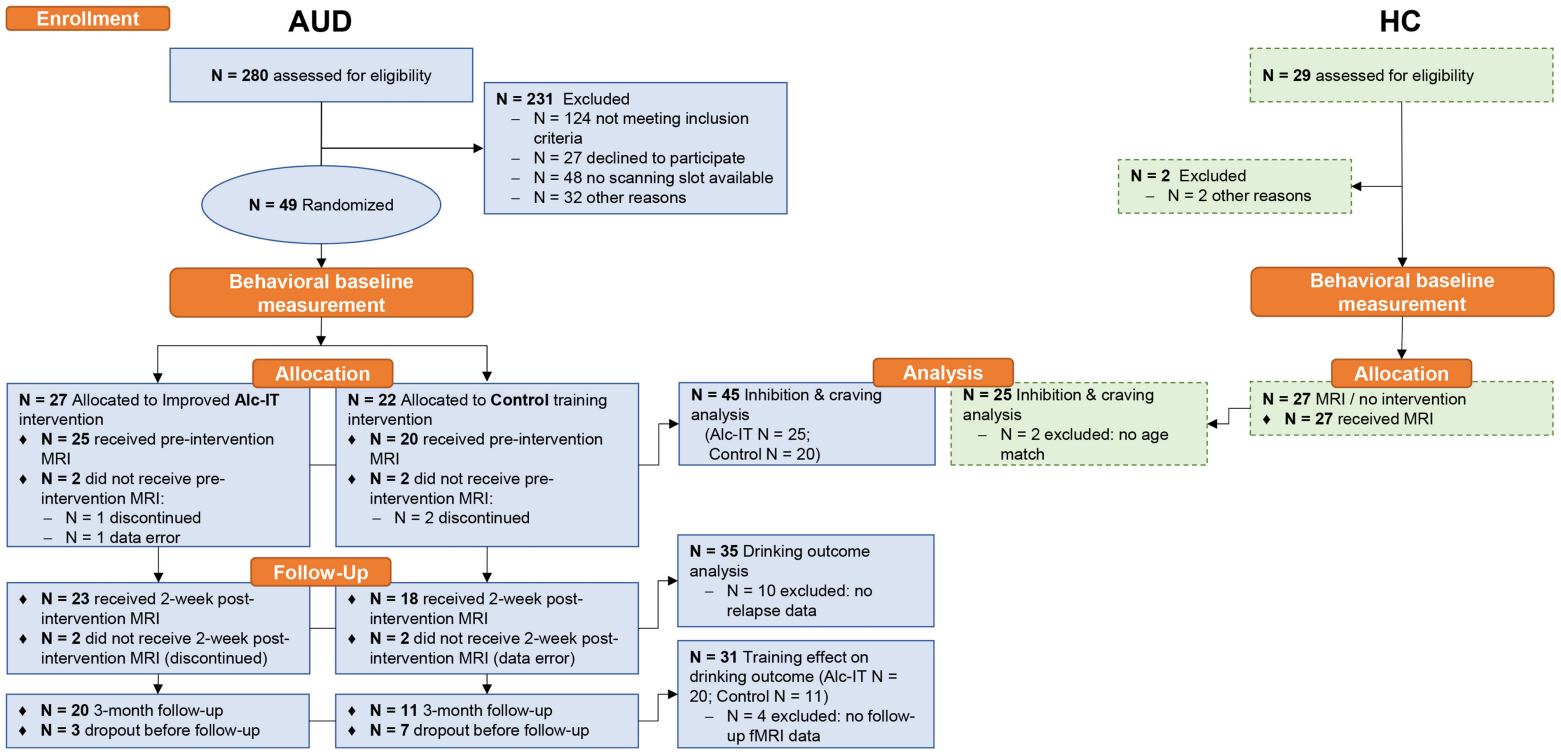
Study design of the GNG-fMRI sub-study within the INTRA project. Dashed box contours indicate data that is shown in Supplementary Material. Alc-IT, alcohol-specific inhibition training; AUD, alcohol use disorder; GNG, Go-NoGo task; HC, healthy controls; MRI, magnetic resonance imaging; N, sample size.

All 49 patients were attending a 12-week abstinence-oriented residential treatment program for AUD in a specialized treatment center in Switzerland (Clinic Suedhang). Inclusion criteria were 18–61 years of age, main diagnosis of AUD according to Diagnostic and Statistical Manual of Mental Disorders, Fifth Edition [DSM-5; (48)], right-handedness and abstinence from alcohol for at least 4 weeks prior to MRI measurement. Exclusion criteria were other severe substance use disorders [except nicotine; Drug Use Identification Test DUDIT ≥25 per substance, (49)], current medical conditions preventing participation (e.g., acute infectious disease), diagnosed neurocognitive disorders (e.g., Korsakoff syndrome), contraindications to perform an MRI or inability to read and understand the participant's information. A control group was recruited including 27 right-handed healthy adults. Low scores of psychopathology [Brief Symptom Check List, BSCL (50, 51) GSI_t−value_ ≤ 63] as well as non-problematic drinking behavior [Alcohol Use Disorders Identification Tests, AUDIT (52) < 8; Alcohol Use Disorder Scale, AUD-S (53) < 2] were inclusion criteria. Exclusion criteria were current or past substance use or disorder [Drug Use Disorders Identification Test, DUDIT (54) < 8 per substance, except nicotine], current psychiatric diagnosis or treatment, and other neurocognitive complications. All participants provided written informed consent and received a reimbursement of 50 Swiss Francs for participation. The main study was approved by the local ethics committee (KEK-number: 2016-00988) and registered at ClinicalTrials.gov (NCT02968537) and the Swiss National Clinical Trials Portal (SNCTP000002043). For more details on procedure, tasks, materials, and questionnaires used in the main study, see Tschuemperlin et al. (47). Three patients discontinued before the first MRI measurement and one had to be excluded because of technical problems during fMRI measurement scan, leading to a final analytic sample of *N* = 45 patients and *N* = 25 healthy controls for the analysis on alcohol-specific inhibition. See Figure 1 for an overview of analytic sample sizes for the different analyses. Detailed sample description is shown in Table 1.

**Table 1:** Descriptive statistics and clinical scores stratified by study groups.

	**AUD Alc-IT** **(*n* = 25)**	**AUD Control** **(*n* = 20)**	**HC** **(*n* = 25)**	
	**Mean (std. dev.)**	**Mean (std. dev.)**	**Mean (std. dev.)**	***p***
Age, years	43.6 (10.4)	43.1 (8.2)	37.4 (12.7)	0.13
Gender (F/M)	9/16	7/13	11/14	–
Education, years	14.5 (3.7)	13.3 (3.1)	16.4 (3.1)^a^	0.004^**^
Employment, yes/no	12/13	12/8	23/2	–
Smoking tobacco, yes/no	18/7	12/8	–	–
Nr. of detox	3.0 (3.0)^b^	3.2 (2.9)^c^	–	1.0
Years of probl. drinking	13.3 (14.1)^f^	11.9 (9.7)^g^	–	0.88
AUDIT	24.6 (6.9)	24.9 (7.5)	–	0.91
BSCL GSI	1.3 (0.8)	1.1 (0.6)	0.17 (0.17)	<0.001^***^
BDI II	14.8 (10.3)^h^	16.7 (9.9)^i^	–	0.67
BAI	8.5 (11.5)	9.1 (7.8)	–	0.91
OCDSimp	13.8 (3.5)	14.1 (3.7)	1.9 (1.8)	<0.001^***^
OCDScog	10.0 (4.7)	9.8 (5.1)	0.1 (0.3)	<0.001^***^
OCDSsum	23.8 (7.5)	23.9 (8.1)	2.0 (1.9)	<0.001^***^
PDA Δ	72.5 (20.8)^d^	56.1 (45.9)^e^	–	0.28
AUD-S	28.1 (7.5)	30.0 (7.9)	–	0.49

a *n = 24,*

b *n = 12,*

c *n = 11,*

d *n = 22,*

e *n = 15,*

f *n = 19,*

g *n = 17,*

h *n = 23,*

i *n = 20.*

** *p < 0.01;*

*** *p < 0.001.*

*Alc-IT, alcohol-specific inhibition training; AUDIT, Alcohol Use Disorder Identification Test; AUD-S, Alcohol use disorder - Scale, BSCL-GSI, general symptom index of the Brief Symptom checklist; Control, Control training; F, Female; HC, healthy controls; M, Male; Nr. of detox, Number of prior detoxifications; OCDS, Obsessive-Compulsive Drinking scale. OCDSimp: subscale capturing compulsive drinking; OCDScog: subscale capturing cognitive aspects of craving; OCDSsum: overall score of transsituational craving; PDA Δ, Difference in Percentage of days abstinent between baseline and 3-months follow-up; std. dev., standard deviation; TTFD, Time to first drink in days after discharge from inpatient treatment; 3m-FU, Assessment 3 months after discharge from inpatient treatment; WHOQOL, WHO Quality of Life Scale (47)*.

### Questionnaires and Interviews

At baseline, a trained study member verified AUD diagnosis with the Diagnostic Expert System for Psychiatric Disorders [DIA-X, the AUD part adapted to DSM-5, (55)]. A questionnaire battery assessed self-rated AUD symptoms [Alcohol Use Disorder-Scale, AUD-S, adapted to DSM-5, (53)], alcohol-related problems [Alcohol Use Disorders Identification Tests, AUDIT (52)], general psychopathological symptoms [Brief Symptom Check List, BSCL (50, 51)], depressivity [Beck Depression Inventory, BDI-II (56)], anxiety [Beck Anxiety Inventory, BAI (57)] alongside demographics, socioeconomical data and other relevant clinical characteristics [see Tschuemperlin et al. (47)] for a complete description of measures. Also included in this questionnaire battery was the Obsessive Compulsive Drinking Scale [OCDS-G (58)], which assessed transsituational craving in the prior week. The OCDS is a reliable and widely used measure of transsituational craving that has been validated in populations similar to the current sample (58–61). Next to an overall score and a cognitive subscale, the OCDS provides a subscale capturing the behavioral aspects of craving such as drinking compulsions. This subscale (OCDSimp) has been used in prior similar studies and is also used here to operationalize craving in order to allow for optimal comparability and integration of the present study in literature.

Assessment of alcohol consumption was assessed at baseline (assessing drinking in the 90 days prior to detoxification entry) and 3-month follow-up (assessing drinking in the 90 days following treatment discharge) with the TLFB interview (62). From TLFB data the percentage of days abstinent (PDA) was computed for baseline and 3-months follow-up after correction for days spent in a protected environment (i.e., a residential treatment center or a somatic hospital). The change in PDA [PDA Δ, computed as PDA_(3−monthfollowup)_ – PDA_(Baseline)_] was used in those fMRI analyses, which investigated the relation between the neurophysiology of alcohol-specific inhibition and drinking outcomes.

### Go-NoGo (GNG) Event-Related fMRI Task

**Figure 2:**
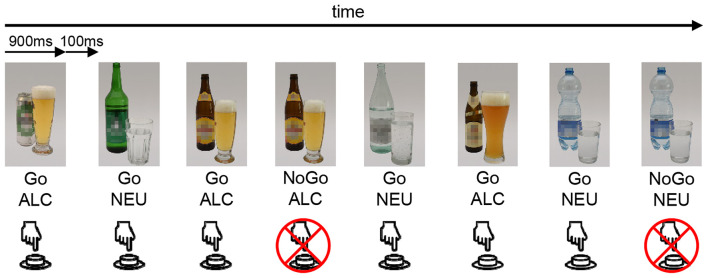
Schematic illustration of the GNG event-related fMRI task. The stimuli are either an alcoholic bottle with a glass (ALC) or a neutral (i.e., water) bottle with a glass (NEU). This is an example of the beer set, whereas depending on the participant's preference, also wine or spirits sets were available. Participants were instructed to press a button whenever a stimulus appeared (Go), unless the exact same stimulus was shown twice successively (NoGo).

The task used in this study to assess BOLD-responses of alcohol-specific and neutral inhibition was equal to the task in Batschelet et al. (43). Building on a classical, neutral Go-NoGo task (36, 63), this task was developed to investigate response inhibition in a neutral as well as in an alcohol-related context (see Figure 2). Participants were presented with a series of pictures on a computer screen and were instructed to press a button as soon as possible whenever the presented picture changed (Go trial), but to withhold that response, when the same picture was repeated (NoGo trial). Participants were instructed to answer as fast and as accurately as possible. Stimuli were tailored according to the personal preference of the participants by drawing from three sets of stimulus material (beer, spirits, or wine). Each set consisted of eight photographs of alcoholic (ALC) drinks and eight photographs of neutral (NEU) drinks (mineral water). All pictures were taken with a high-resolution camera in standardized lighting conditions (47). Each photograph was displayed 60 times to the participant, 52 times in a Go condition, and eight times in a NoGo condition. This sums up to 960 trials, comprising 416 Go_ALC_, 416 Go_NEU_, 64 NoGo_ALC_, and 64 NoGo_NEU_ trials, leading to a Go/NoGo ratio of 6.5. The trials were presented in a pseudorandomized order with a mean of 7.5 Go-trials (i.e., 7.5 s) between two NoGo-trials. The task was subdivided into two blocks with a break of 1 min and 1 s. Photographs were displayed for 900 ms with a 100 ms inter-stimulus interval blank screen. The GNG-task was created and response data was logged with the E-Prime 2.0 software (Psychology Software Tools Inc., Pittsburgh, PA, USA).

### MRI Data Acquisition

Functional and anatomical MRI data acquisition was conducted at the University Hospital of Bern, using a Siemens Magnetom Prisma scanner with 3 Tesla magnetic field strength and a head coil with 64 channels. For functional image acquisition during the above described GNG-task, a multi-band echo planar imaging (EPI) sequence was run (TR/TE = 1,300/37 ms; 60 slices; slice thickness = 2.2 mm; voxel-size 2.2 × 2.2 × 2.2 mm; FOV = 230 × 230 mm; matrix size = 105 × 105). For subsequent image distortion correction, a b0 protocol was run to acquire 4 field map images (2 phase/amplitude each) with the same image geometry as in the EPI-sequence, with TR/TE1/TE2 = 591/4.92/7.38 ms. Anatomical images were obtained using an MP2RAGE sequence (TR/TE = 5,000/2.98 ms; inversion time T1/T2 = 700/2,500 ms; 256 slices; slice thickness = 1.0 mm; voxel-size 1 × 1 × 1 mm; FOV = 256 × 256 mm; matrix size = 256 × 256). Note that, while the combination of a fast event-related task design with BOLD-fMRI is not optimal, such a combination has—despite the drawbacks and a reduced signal-to-noise-ratio—yielded important insights into the neural basis of inhibition in prior studies (36, 63).

### Pre-processing and Analysis of Functional MRI Images

#### Pre-processing

Task-fMRI images were preprocessed using the routines implemented in SPM12. Initially, the origins of all functional and anatomical images were reoriented to the anterior commissure. The field map images were used to construct the voxel displacement map for unwarping, which was applied after realignment that involves participant's motion correction. Subsequently, slice time correction was run as well as coregistration of the functional images to the anatomical image. A brain tissue segmentation was performed to obtain forward deformation fields for the image normalization procedure, which transformed the images to the MNI standard space. Finally, all functional images were smoothed using a 3D-Gaussian Kernel with 6 mm^3^ FWHM.

#### First-Level Analysis

In order to extract functional activation images for each GNG stimulus condition, contrast images at the subject level were generated (e.g., 1st-level analysis). For this purpose, the preprocessed images were entered to a general linear model (GLM). In detail, a design matrix was constructed with the predefined GNG task events consisting of stimulus type (ALC, NEU), response type (NoGo, Go), and participant's response accuracy (correct, error). We used the canonical hemodynamic response function (HRF) as a basis function that was convoluted with the onsets of the resulting eight event types (NoGo_ALC_correct_, NoGo_NEU_correct_, Go_ALC_correct_, Go_NEU_correct_, NoGo_ALC_error_, NoGo_NEU_error_, Go_ALC_error_, and Go_NEU_error_). The six individual motion parameters derived from realignment were entered as regressors of no interest to the design matrix. Finally, the GLM was estimated. For calculation of contrast images, only parameter estimates of regressors corresponding to event types with correct participant responses were included, since this study focused exclusively on successful inhibition. Thus, the following four contrasts were computed: (NoGo_ALC_ + NoGo_NEU_) > (Go_ALC_ + Go_NEU_), NoGo_ALC_ > Go_ALC_, NoGo_NEU_ > Go_NEU_, (NoGo_ALC_ > Go_ALC_) > (NoGo_NEU_ > Go_NEU_).

#### Second-Level Analyses

These first-level contrast images were used to calculate random effects at the group or second level. First, we investigated whether neuronal activation during alcohol-specific inhibition increases with craving (assessed with OCDSimp). To this end, a whole-brain linear regression was performed with NoGo_ALC_ > Go_ALC_ with OCDSimp as the covariate of interest. A second linear regression using NoGo_NEU_ > Go_NEU_ and OCDSimp as the covariate was performed to tests whether results were specific for alcohol-related inhibition. In order to have this analysis encompass a broad spectrum of craving levels, healthy controls were included in this analysis in addition to patients with AUD (see right panel of Figure 1), leading to an analytic sample of *N* = 70. For this analysis fMRI-data from the pre-training session was used.

Second, a planned contrast (NoGo_ALC_ > Go_ALC_) > (NoGo_NEU_ > Go_NEU_), which isolates alcohol-specific inhibitory activation, was used for exploring whether the neurophysiological signature of alcohol-specific inhibition is predictive for drinking outcomes. More detailed, the individual planned contrast whole brain images (from the pre-training fMRI session) of *N* = 35 patients with AUD were used in a whole brain linear regression with the drinking outcome (indicated by PDA Δ) as the covariate of interest. This analysis thus used the fMRI data from the pre-training session to predict drinking outcome at 3-months follow-up. For this analysis, only gray matter voxels were used by applying a binary mask derived from the mean over all individuals' MP2RAGE gray matter segmented image. Brain regions yielded by this analysis as reflecting a neural correlate of alcohol-specific inhibition which is predictive of relapse, will be used as regions of interest (ROIs) in the third analysis (see below).

Third, to investigate whether the thus functionally defined, relapse-predicting, alcohol-specific inhibitory activation can be altered through training, the MNI-coordinates of potential effects yielded by the second whole-brain analysis served as regions of interest (ROIs). From these ROIs, beta-values were extracted for each subject, for which a complete dataset was obtained. The analytic sample of this third analysis consisted of *N* = 31 patients with AUD, for which pre-training fMRI data, post-training fMRI data, and drinking outcome data at 3-months follow-up was available. The beta-value analysis was applied to investigate whether the magnitude of alcohol-specific inhibitory activation predictive for drinking outcome was modulated by the training. Therefore, a 2 × 2 × 2 × 2 repeated-measures ANOVA was conducted using the beta-values of the potential ROIs as dependent variable and featuring the between-subject factor training (Alc-IT/Control) and the three within-subject factors response type (NoGo/Go) × stimulus type (ALC/NEU) × pre-post (pre-training/post-training). A significant 4-way interaction was needed to confirm a training-effect on alcohol-specific inhibition in the potential ROI that is predictive for drinking outcome.

For completeness, a basic analysis used the (NoGoALC + NoGoNEU) > (GoALC + GoNEU) contrast (pre-training images only) in one-sample *t*-tests run separately for the AUD and HC groups with the aim of contributing to the research on general inhibition in AUD. As general inhibition was not the main focus of this study, these results can be found in the Supplementary Figures 1, 2.

### Statistical Analyses

**Figure 3:**
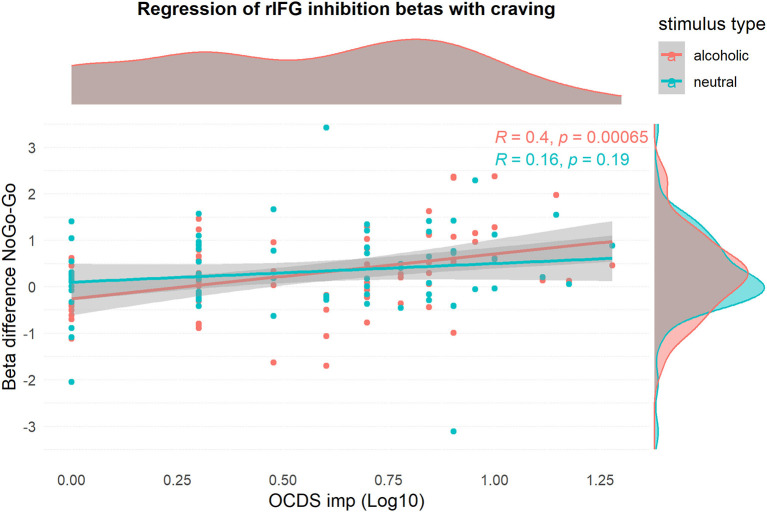
Visualization of the relationship of rIFG beta-values and log10-transformed OCDSimp scores, stratified for stimulus type. The graph confirms that craving has a positive relationship with inhibition activation during alcohol-related inhibition only.

**Figure 4:**
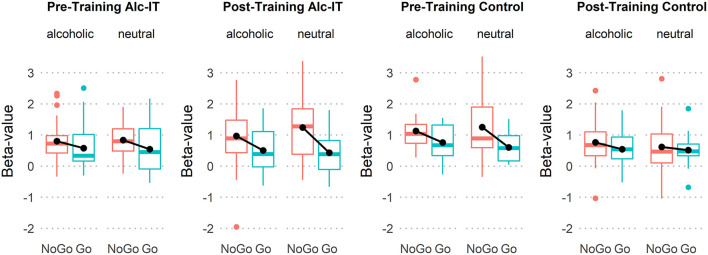
Boxplots indicating beta-value differences of response type (NoGo in red, Go in green), stimulus type, pre- and post-training, and training type. Black lines connecting black dots highlight mean-differences in inhibition activation.

Statistical Package for Social Sciences (SPSS, version 28, IBM Corp., Armonk, NY, USA) was used to assess group differences of demographics and clinical scores. All variables except the BDI II score were non-normally distributed. Therefore, to test differences between the two AUD subgroups, non-parametric Kolmogorov-Smirnov tests were run. For differences over all three groups, non-parametric Kruskal-Wallis tests were computed. Behavioral GNG data [reaction times (RTs), errors of commission (EOC), errors of omission (EOO)] were non-normally distributed and therefore analyzed with ANOVA-type non-parametric statistics using the nparLD package in R (64). This analysis was run twice, first including factors AUD and HC as group variable, and second with factors Alc-IT and control training as group variable. For statistical inference involving voxel-wise fMRI-data (linear regression with NoGo_ALC_ > Go_ALC_ and OCDSimp, linear regression with NoGo_NEU_ > Go_NEU_ and OCDSimp, linear regression with planned contrast and PDA Δ, and one-sample *t*-tests with NoGo > Go), SPM12 routines were applied with a family-wise error (FWE) correction at critical *p*-value threshold of 0.05, with a cluster size of 0. The repeated-measures ANOVA involving beta-values of the ROI was computed with SPSS. Anatomical brain and activation illustrations were created using the MRIcroGL software (https://www.nitrc.org/projects/mricrogl/). Finally, statistics necessary to produce illustrations (Figures 3, 4) were calculated with R (v4.1.0).

## Results

### Descriptive and GNG Behavioral Statistics

While the three study groups did not differ in age, the HC group showed a higher education level than the AUD group. As expected, the HC group scored lower in BSCL GSI, OCDSimp, and OCDSsum scores than the AUD group. All group means as well as group statistical *p*-values can be found in Table 1. Important for comparisons between the two AUD training groups, no differences were found in any demographic or clinical variable. Underlining the validity of the OCDSimp scale, which was used to operationalized craving, correlations between OCDSimp and a visual analog scale to assess craving are shown in Supplementary Figure 3.

Analyzing the GNG behavioral data from the pre-training measurement, both AUD and HC made more EOCs with NEU trials than ALC trials as confirmed by a significant main effect of stimulus type [ANOVA-type-statistics (ATS)_(1)_ = 7.25, *p* = 0.007]. EOCs, the main indicator of inhibitory performance, did however not differ between patients with AUD and HCs. The RTs of EOC were significantly shorter in NEU than ALC [ATS_(1)_ = 5.15, *p* = 0.023], and shorter in HC compared to AUD [ATS_(1)_ = 4.92, *p* = 0.027], although the two-way interaction was not significant [ATS_(1)_ = 0.36, *p* = 0.548]. Concerning the number of EOOs the AUD group performed worse than the HC group [ATS_(1)_ = 5.48, *p* = 0.019]. Again, the interaction was not significant [ATS_(1)_ = 0.16, *p* = 0.686]. RTs in correct Go trials were shorter in HC compared to AUD [ATS_(1)_ = 7.40, *p* = 0.007], with no significant stimulus type × group interaction [ATS_(1)_ = 0.32, *p* = 0.573].

The next analysis compared the patients' GNG behavioral data between the two training groups (Alc-IT, Control) and both timepoints (pre taining/post training) and stimulus types (ALC, NEU). This analysis indicated that numbers of EOC were higher in NEU than ALC [ATS_(1)_ = 12.38, *p* < 0.001]. The two-way [pre/post-training × training type: ATS_(1)_ = 4.94, *p* = 0.026] and three-way interaction (stimulus type × pre/post-training × training type) were also significant [ATS_(1)_ = 6.11, *p* = 0.013]. Follow-up analyses of these interactions showed that only in the NEU condition, but not in the ALC condition, the two-way interaction (pre/post-training × training type) was significant [NEU stimulus type: ATS_(1)_ = 11.18, *p* < 0.001; ALC stimulus type: ATS_(1)_ = 0.35, *p* = 0.555]. Subsequent simple effects analyses indicated that EOCs in the NEU condition only decreased in the control training [pre/post-training effect: ATS_(1)_ = 15.39, *p* < 0.001], and not the Alc-IT training type [ATS_(1)_ = 1.85, *p* = 0.174]. When analyzing the two-way interaction of stimulus type and pre/post-training separately for each training group, this interaction was not significant in both groups [Alc-IT: ATS_(1)_ = 2.71, *p* = 0.100; Control: ATS_(1)_ = 2.58, *p* = 0.108].

Such effects were not found in the RTs of EOC [all effects ATS_(1)_ < 1.55, *p* > 0.21]. In the number of EOO, main effects of pre/post-training [ATS_(1)_ = 4.62, *p* = 0.032], and stimulus type [ATS_(1)_ = 12.61, *p* < 0.001] were found. In particular, the number of EOO was higher pre- compared to post-training, and higher for ALC compared to NEU. Finally, correct response RTs in the Go condition were shorter post than pre training RTs [ATS_(1)_ = 19.53, *p* < 0.001]. Means and standard deviations of the analyzed GNG behavioral variables can be found in Supplementary Tables 1, 2.

### Does Neuronal Activation Related to Alcohol-Specific Inhibition Increase With Craving?

#### Linear Regression of Alcohol-Specific Inhibitory FMRI Data With OCDSimp

**Figure 5:**
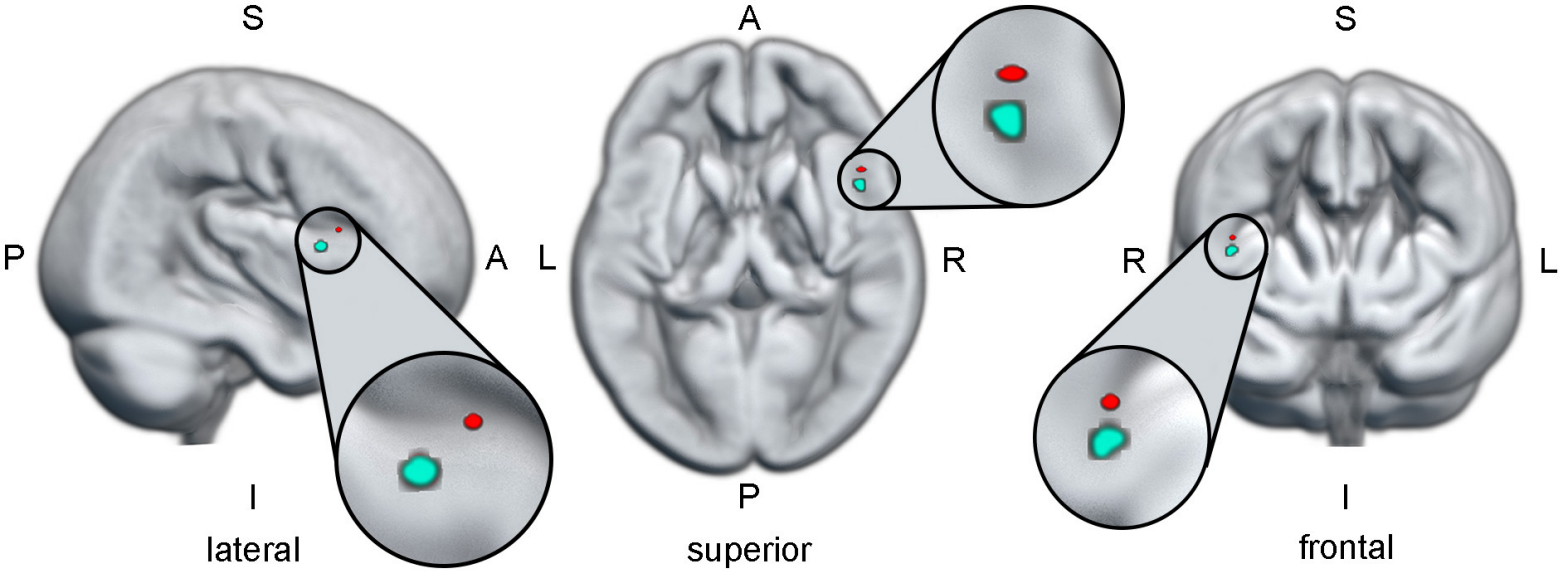
The red dot indicates the region in the right inferior frontal gyrus, where a higher activation during alcohol-related inhibition is predictive for higher craving. The cyan dot indicates the region in the right inferior frontal gyrus, where a higher activation during alcohol-specific inhibition is predictive for a better drinking outcome. The anatomical brain render used for this illustration is based on the mean normalized gray matter image of all participants included in this study. All three views were cut from the surface to the localization of the cyan ROI. Since the red ROI is on the same pane as the cyan ROI only in the lateral view, the red ROI should be viewed as “hovering in the air” in the superior and frontal views. A, anterior; I, inferior; L, left; P, posterior; R, right; ROI, region of interest; S, superior.

This analysis was performed to identify brain regions, where alcohol-specific inhibitory activation increased with craving. Using the NoGo_ALC_ > Go_ALC_ contrast, a small ROI was found in the right inferior frontal gyrus, which showed a positive relationship between brain activation and OCDSimp scores (Table 2, Figure 5). This indicates that alcohol-related inhibitory brain activation in rIFG was higher in those subjects reporting high craving as compared to subjects with low craving. Contrary to that, the NoGo_NEU_ > Go_NEU_ contrast yielded no significant clusters (Supplementary Figure 4A). Using the beta-values of the rIFG ROI, Figure 3 illustrates that the correlation of craving and inhibitory brain activation was specific for alcohol-related stimuli. *Post-hoc* analyses inspecting the correlation between inhibitory activation in the IFG ROI and craving separately for the patient group replicated this result and indicated that for patients, craving levels correlated significantly with alcohol-related rIFG activation (Supplementary Figure 4B).

**Table 2:** SPM output listing significant cluster with statistics and MNI-coordinates of the regression analyses investigating gray matter whole brain relationships of inhibition activation and craving as well as drinking outcome.

	**Peak-level**					
**Atlas label^a^**	***p*_FWE-corr_**	***T***	***k*_E_**	**mm**	**mm**	**mm**
**Alcohol inhibition/craving**						
rIFG, pars opercularis	0.032	4.87	4	48	18	12
**Planned contrast/drinking outcome**						
rIFG, pars opercularis	0.005	6.73	9	48	12	8
rIFG, pars opercularis	0.021	6.10	2	62	16	16

a *Harvard-Oxford Cortical Structural Atlas.*

*kE, cluster extend; pFWE-corr, p-value yielded by analysis using family-wise error correction; rIFG, right inferionr frontal gyrus*.

### Is the Neurophysiological Signature of Alcohol-Specific Inhibition Related to Drinking Outcomes?

#### Linear Regression of Planned Contrast FMRI Data With PDA Δ

This analysis was conducted to identify possible regions of interest, where the activation during alcohol-specific inhibition (as isolated in the planned contrast) predicts drinking outcome (as indicated by PDA Δ) in the patients with AUD. Table 2 lists the statistics and MNI coordinates of two significant clusters that showed a positive relationship between alcohol-specific inhibitory activation and PDA Δ. No significant clusters were found with a negative relationship. In accordance with Eklund et al. (65) we report peak-level statistics rather than cluster-level statistics. Hence, higher alcohol-specific inhibition activation in the regions found in the right inferior frontal gyrus is predictive for a better drinking outcome. While both regions are comparably small in cluster extent, we limited our *post-hoc* analysis only on the larger of the two regions. Figure 5 illustrates the anatomical localization of the larger region superimposed on the mean gray matter image of the study cohort.

### Can the Neurophysiological Signature of Alcohol-Specific Inhibition Be Altered Through Alcohol-Specific Inhibition Training?

#### ANOVA Using RIFG Beta Values and Assessing Potential Training Effects

This analysis aimed at investigating whether Alc-IT had a beneficial effect on the alcohol-related inhibitory activation in the rIFG ROI identified in the previous analysis. Figure 4 displays boxplots providing an overview of the rIFG beta values for each response type, stimulus type, pre-training and post-training session, and training type. The 2 × 2 × 2 × 2 repeated-measures ANOVA yielded a main effect of inhibition [*F*_(1, 29)_ = 14.7, *p* < 0.001, *η*^2^ = 0.336], showing higher activation in NoGo compared to Go trials. Moreover, a significant three-way interaction of response type × pre- post-training × training type [*F*_(1, 29)_ = 4.4, *p* = 0.045, *η*^2^ = 0.132] indicated that Alc-IT has an effect on inhibition activation in general, but not specifically on alcohol-related inhibition. This interaction can be seen in Figure 4 when inspecting the slope of the black lines reflecting the magnitude of inhibitory activation. Specifically, the slope steepness seems to increase from pre-training to post-training in Alc-IT, whereas it appears to decrease in control training. However, the target four-way interaction was not significant [*F*_(1, 29)_ = 1.6, *p* = 0.213, *η*^2^ = 0.053], opposing to our hypothesis of a beneficial effect of Alc-IT on alcohol-specific inhibition (at least in the ROI analyzed).

## Discussion

Analyzing fMRI data collected during an alcohol-related GNG task, this study set out to investigate whether neurophysiological correlates of alcohol-specific inhibition (I) vary with craving (see Section Does Neuronal Activation Related to Alcohol-Specific Inhibition Increase With Craving?); (II) are related to drinking outcomes in AUD (see Section Is the Neurophysiological Signature of Alcohol-Specific Inhibition Related to Drinking Outcomes?) and (III) can be altered through alcohol-specific inhibition training (see Section Can This Relapse-Predicting Neurophysiological Signature of Alcohol-Specific Inhibition Be Altered Through Alcohol-Specific Inhibition Training?).

### Does Neuronal Activation Related to Alcohol-Specific Inhibition Increase With Craving?

In a small cluster of the right inferior frontal gyrus (rIFG), the neurophysiological correlates of alcohol-related (but not neutral) inhibition increased with craving, indicating that brain activation during alcohol-related inhibition was higher in those subjects, which experienced higher levels of craving. This finding extends earlier reports of craving being linked to enhanced neurophysiological activation in cingulate areas during alcohol-specific inhibition (31, 42) to the rIFG. While it supports the general idea of higher craving being linked to enhanced neurophysiological activation during successful alcohol-specific inhibition, one must acknowledge that the specific locations do not align across studies. This might be due to differences in stimulus material (31, 42) or due to EEG (42) and fMRI measuring non-overlapping aspects of the neuronal activation.

The rIFG is part of the cognitive control network (66, 67) and particularly the pars opercularis (68) has been shown to be selectively activated by tasks requiring response inhibition (22, 23). Recent reviews of neurofunctional networks involved in addictive disorders feature the IFG as a central node of a dysregulated inhibitory control network in substance use disorder (SUD) (4, 5). In patients with SUD, higher rIFG activation was furthermore linked to decreased attentional impulsiveness (69) and to a better ability to ignore drug-related stimuli during a working memory task (70), supporting its role in suppressing cue-induced responses. Higher rIFG activation during successful alcohol-related inhibition, which we observed in those patients reporting higher craving, might thus be indicative of additional neuronal resources being necessary in order to control responses in the face of highly salient and reward-predicting stimuli.

### Is the Neurophysiological Signature of Alcohol-Specific Inhibition Related to Drinking Outcomes?

Again in the rIFG, neurophysiological activation during alcohol-specific inhibition was related to a better outcome, as indicated by a higher percentage of days abstinent at 3-month follow-up. Those patients displaying a higher activation difference in the rIFG for alcohol-related (as compared to neutral) inhibition reported more days abstinent at 3-months follow-up. The outcome was thus better in those patients, who managed to recruit enhanced neuronal resources during inhibition when the inhibitory system had to oppose cue-induced appetitive processes.

As summarized above, the rIFG is closely linked to effective inhibition and has been linked to response suppression in the face of craving and cue-induced reactions (4, 70). Our results thus suggest that enhanced inhibitory rIFG activation in the face of alcohol-related stimuli might enhance the chance to inhibit potential drinking urges or automatized drinking habits and thus fosters abstinence in patients with AUD. Such an interpretation is in line with an earlier study indicating that the neurophysiologic correlate of alcohol-specific inhibition, as measured with event-related potentials, predicts relapse in AUD (43). More closely related to the rIFG, an earlier fMRI study in patients with AUD also linked stress-induced activation in the right ventrolateral PFC, which includes the rIFG, to alcohol use in a 90-day follow-up period [(71), note however that in the same study, a linkage between alcohol-induced cue reactivity and relapse could not be deteccted].

Taken together, the present results indicate that higher rIFG activation is necessary to inhibit responses to alcohol-related cues when craving is high. In line with this, as most patients with AUD probably experience situations with high craving when returning to everyday life after residential treatment, enhanced IFG activation during alcohol-specific inhibition predicts a better outcome at 3-months follow-up.

### Can This Relapse-Predicting Neurophysiological Signature of Alcohol-Specific Inhibition Be Altered Through Alcohol-Specific Inhibition Training?

A training effect on the neurophysiological correlate of alcohol-specific inhibition in the rIFG could not be observed; rIFG activation was not modulated by patients engaging in alcohol-related inhibition training (Alc-IT) vs. an unspecific inhibition training (control). Thus, we cannot conclude that this neurophysiological correlate, which—according to the analyses reported above—varies with craving and predicts drinking outcome, is affected by the alcohol-specific inhibition training. In that respect, our results differ from reports on another cognitive training intervention, approach bias training, where training effects on brain activity could be observed, albeit in ROI analyses focusing on other brain regions and during different tasks (72, 73). One possible explanation is the limited statistical power in this study, which was smallest for this third research question due to the complex design and the restriction to those patients providing follow-up data. Furthermore, it might be that neurophysiological effects of Alc-IT are better detectable with neurophysiological methods allowing for a higher temporal resolution (74). Also, we focused our analyses of training effects on a ROI in the rIFG, which was functionally defined as the region in which we found alcohol-specific inhibitory effects related to relapse. This analytic approach was conceptually motivated by prior research supporting the hypothesis that Alc-IT enhances alcohol-specific inhibition and might reduce relapse risk (3). However, it is also conceivable that Alc-IT produces training effects centered in other regions of the brain. Such effects would have been overlooked by the present ROI analyses and might be too small to be detected in a whole brain analysis (which we conducted *post-hoc* with no significant results). To be complete, one should mention that the neurophysiological correlate of general (but not alcohol-specific) inhibition was differentially affected in the group receiving Alc-IT when compared to control training. As there was however no theoretical justification for a ROI-analysis focusing on this three-way interaction in the rIFG, this finding has to be seen as highly exploratory.

As a general limitation, one might argue that the effects reported above are very small. The resulting clusters were smaller than 10 voxels in extent. Besides the FWE-correction, no additional cluster size threshold was applied. None of the three clusters reported in Table 2 would be significant with the even more conservative topological FDR-correction, and only the largest cluster would remain significant using the TFCE-method (*p*_FWE−corr_ = 0.030; *T* = 5.95; *k*_E_ = 3; x = 48 mm; y = 12 mm; z = 8 mm). Besides the fact that the application of the FWE-correction used here is a common way to minimize type-I errors, one should however take into account two important aspects: (I) Alcohol-specific inhibitory activation, as we attempted to isolate it in this study, is reflected in a highly specific effect. Our analyses thus had to concentrate on those inhibitory sub-processes that vary with the context in which inhibition had to be carried out. On the one hand, such a highly specific effect might be represented on smaller sub-regions. On the other hand, such an effect might also be harder detectable in a noisy signal such as BOLD. One might therefore consider the application of higher field strengths (e.g., 7 Tesla), where a higher signal should be expected and where the higher spatial resolution might also allow a better investigation of subregions of IFG. (II) The spatial extend of the rIFG clusters amounted to 32 and 72 mm^3^, respectively. With a neuronal density of several thousand per mm^3^, we should not preclude that activation of such a specific cognitive process is possible within these small regions, unless there exists indication that it is impossible. In addition, it appears unlikely that two different whole-brain analyses yielded exclusively two highly proximal clusters in a region central to inhibition (66, 68) purely by chance. Therefore, in order to avoid not only type I, but also type II errors (75), the present results should be considered as neurophysiologically meaningful, which should of course be corroborated by replication in future studies.

Another limitation concerns the task design. Compared to earlier studies (31, 42), the present task employed a limited number of different alcohol-related pictures. This led to each picture being displayed more often than in earlier studies and might have facilitated habituation, which might have dampened effects related to alcohol-specific inhibition.

While the behavioral performance on the Go-NoGo task was not the focus of this study, two aspects are still worth noting. A first aspect concerns the fact that in the present study, patients with AUD did not display an inhibitory performance deficit (as indicated by errors of commission (EOCs) during NoGo trials). This is inconsistent with the overall pattern yielded by meta-analyses summarizing behavioral studies on inhibitory performance (8, 9) and with some studies on neurophysiological correlates of inhibition in substance use disorder (36, 37). However, it is in line with other neurophysiological studies on inhibition in substance use disorder (25, 26, 29, 34, 76). In the literature, these inconsistencies are discussed with respect to the stimulus material and task context (alcohol-related or neutral)(4), to methodological details (17) as well as to differences in the specific stud samples ability recruit the necessary additional neuronal resources to achieve a comparable performance (5, 31).

A second aspect concerns the fact that no effect of the Alc-IT training intervention on alcohol-related EOCs could be detected. As studies on behavioral effects of cognitive training interventions are often larger, this might either be due to a limited power or to the fact that Alc-IT really did not affect alcohol-related errors of commission.

In summary, the present study corroborated the central role of the rIFG in the inhibitory control network in AUD by indicating that rIFG activation during alcohol-related inhibition is related to craving and drinking outcome. Therefore, as has been proposed before in the context of cocaine addiction (69), future studies might investigate under which circumstances rIFG activation might potentially serve as a biomarker for increased relapse risk.

## Data Availability Statement

The datasets presented in this article are not readily available because no consent for this was collected from patients. Processed data and corresponding processing subroutines can be requested from the corresponding author. Requests to access the datasets should be directed to maria.stein@upd.unibe.ch.

## Ethics Statement

The studies involving human participants were reviewed and approved by the Local Ethics Committee (KEK-number: 2016-00988) and registered at the Swiss National Clinical Trials Portal (SNCTP000002043) and ClinicalTrials.gov (NCT02968537). The patients/participants provided their written informed consent to participate in this study.

## Author Contributions

MG collected the data, conceptualized and performed data and statistical analyses, and wrote the manuscript. LS and FM designed the study and supervised data collection and data curation. RT and HB collected the data. AF conceptualized and performed data and statistical analyses. SS collected the data and conceptualized data analyses. YM supervised data collection and data curation. MS designed the study, supervised data collection and data curation, conceptualized data and statistical analyses, and wrote the manuscript. All authors contributed to discussion and interpretation of the results, revised, and approved the final version of the submitted manuscript.

## Funding

This study was funded by the Swiss Foundation for Alcohol Research (SSA-Nr. 283) and the Swiss National Science Foundation (SNF; No. 105319_159286).

## Conflict of Interest

The authors declare that the research was conducted in the absence of any commercial or financial relationships that could be construed as a potential conflict of interest.

## Publisher's Note

All claims expressed in this article are solely those of the authors and do not necessarily represent those of their affiliated organizations, or those of the publisher, the editors and the reviewers. Any product that may be evaluated in this article, or claim that may be made by its manufacturer, is not guaranteed or endorsed by the publisher.
