# Supplementary material for: Right Inferior Frontal Activation During Alcohol-Specific Inhibition Increases With Craving and Predicts Drinking Outcome in Alcohol Use Disorder
Source: Front Psychiatry. 2022 Jul 1;13:909992. doi: 10.3389/fpsyt.2022.909992 (PMC9283687; doi:10.3389/fpsyt.2022.909992)
Supplement: Supplementary file 1 [file Data_Sheet_1.PDF]

*Supplementary Material for article entitled*

# Right inferior frontal activation during alcohol-specific inhibition increases with craving and predicts drinking outcome in alcohol use disorder

Matthias Grieder[1#], Leila M. Soravia[1,2#], Raphaela M. Tschuemperlin[1,2], Hallie M. Batschelet[1], Andrea Federspiel[1], Simon Schwab[1], Yosuke Morishima[1], Franz Moggi[1°], Maria Stein[1,3*°]

**Supplementary Table 1.** Means and standard deviations of EOC, EOO, and correct Go responses for the AUD and the HC group.

| Response type | Stimulus type | AUD *Mean (SD)* | HC *Mean (SD)* |
|---|---|---|---|
| **Number of errors of commission (EOC):** | | | |
| NoGo | ALC | 13.14 (7.16) | 16.68 (8.41) |
| NoGo | NEU | 15.20 (7.62) | 17.76 (8.14) |
| **RTs of errors of commission (EOC):** | | | |
| NoGo | ALC | 374.03 (74.53) | 335.08 (43.12) |
| NoGo | NEU | 374.88 (56.73) | 344.68 (47.05) |
| **Number of errors of omission (EOO):** | | | |
| Go | ALC | 11.45 (19.34) | 3.68 (3.71) |
| Go | NEU | 9.57 (15.75) | 3.24 (4.10) |
| **RTs of correct Go responses:** | | | |
| Go | ALC | 404.55 (19.34) | 412.32 (3.72) |
| Go | NEU | 406.43 (15.75) | 412.76 (4.10) |

Abbreviations: AUD: Alcohol use disorder. ALC: alcohol-related stimulus. HC: Healthy controls. NEU: neutral stimulus. RT: reaction time. SD: standard deviation

**Supplementary Table 2.** Means and standard deviations of EOC, EOO, and correct Go responses for Alc-IT and control training, both pre- and post-training.

| | | | Alc-IT | Control |
|---|---|---|---|---|
| Response type | Stimulus type | Pre-Post | *Mean (SD)* | *Mean (SD)* |
| **Number of errors of commission (EOC):** | | | | |
| NoGo | ALC | Pre | 12.87 (7.59) | 11.59 (5.15) |
| NoGo | NEU | Pre | 14.70 (9.08) | 15.29 (6.35) |
| NoGo | ALC | Post | 12.70 (8.59) | 10.47 (6.00) |
| NoGo | NEU | Post | 16.13 (8.07) | 12.00 (6.94) |
| **RTs of errors of commission (EOC):** | | | | |
| NoGo | ALC | Pre | 370.86 (75.52) | 374.17 (76.94) |
| NoGo | NEU | Pre | 370.88 (67.70) | 375.93 (40.25) |
| NoGo | ALC | Post | 354.49 (89.32) | 369.55 (58.23) |
| NoGo | NEU | Post | 361.88 (74.63) | 367.54 (66.78) |
| **Number of errors of omission (EOO):** | | | | |
| Go | ALC | Pre | 11.52 (15.85) | 11.53 (24.71) |
| Go | NEU | Pre | 8.61 (11.04) | 10.47 (20.55) |
| Go | ALC | Post | 8.35 (13.32) | 14.24 (40.42) |
| Go | NEU | Post | 5.43 (8.57) | 12.00 (36.90) |
| **RTs of correct Go responses:** | | | | |
| Go | ALC | Pre | 433.49 (62.24) | 420.16 (55.99) |
| Go | NEU | Pre | 435.84 (62.11) | 422.22 (57.57) |
| Go | ALC | Post | 387.38 (73.07) | 399.61 (52.89) |
| Go | NEU | Post | 386.22 (71.22) | 400.45 (52.95) |

Abbreviations: Alc-IT: Alcohol-specific inhibition training. ALC: alcohol-related stimulus. Control: Unspecific control training. NEU: neutral stimulus. Pre: pre-training measurement. Post: post-training measurement. RT: reaction time. SD: standard deviation

**Supplementary Table 3.** Investigation of training effect (Alc-IT/Control) on drinking outcome (PDA baseline/PDA 3-month follow-up).

| **Descriptive statistics** | AUD Alc-IT ($n$ = 20) | AUD Control ($n$ = 11) |
|---|---|---|
| | Mean (std. dev.) | Mean (std. dev.) |
| PDA baseline | 23.7 (22.5) | 13.0 (14.8) |
| PDA 3-month follow-up | 94.8 (7.4) | 82.3 (30.6) |

| **2 × 2 repeated-measures ANOVA** | | |
|---|---|---|
| Effect | $F_{(1,29)}$ | p ($\eta^2$) |
| Time | 189.534 | 3.0028E-14 (0.867) |
| Time × Training | 0.030 | 0.863 (0.001) |
| Training | 4.798 | 0.037 (0.142) |

Note: The upper panel of the table lists means and standard deviations of PDA separately for each training group and time point. The lower panel of the table lists the results of a 2 × 2 repeated-measures ANOVA with the between-subject factor Alc-IT/Control and the within-subject-factor time (pre-training/3-month follow-up). This analysis was conducted using SPSS 28. Note however, that the present study was not designed to detect these training effects on long-term behavioral drinking outcome and is underpowered for such an analysis. Outcomes of a related, larger study [1]are currently under review elsewhere.

Abbreviations: AUD: Alcohol use disorder, Alc-IT: alcohol-specific inhibition training, control: unspecific control training. PDA: Percentage of days abstinent

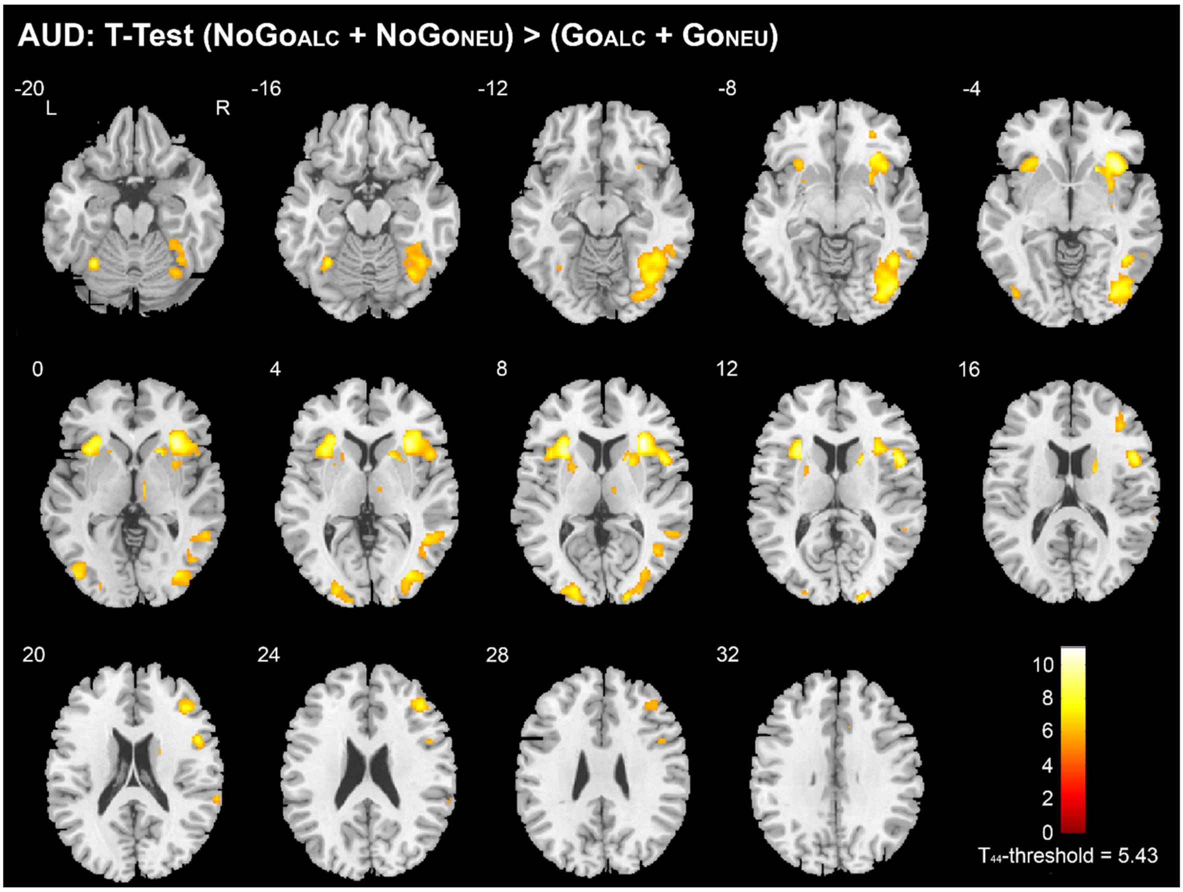


**Supplementary Figure 1.** Mosaic of axial anatomical slices showing the general inhibition activation of the AUD group at the $p_{\text{FWE-corrected}}$-threshold of 0.05.

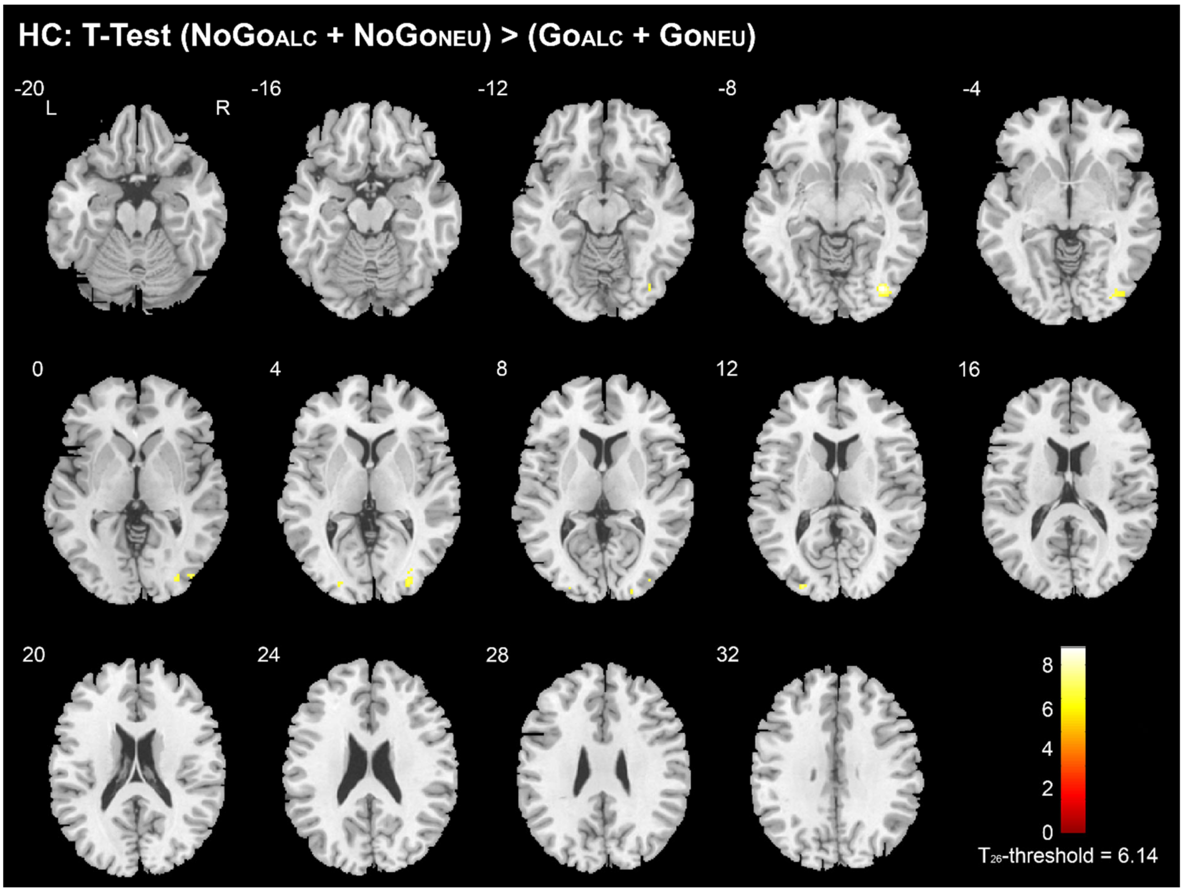


**Supplementary Figure 2.** Mosaic of axial anatomical slices showing the general inhibition activation of the HC group at the $p_{\text{FWE-corrected}}$-threshold of 0.05. Activations can be seen here from pane -12 throughout pane 12 in posterior regions.

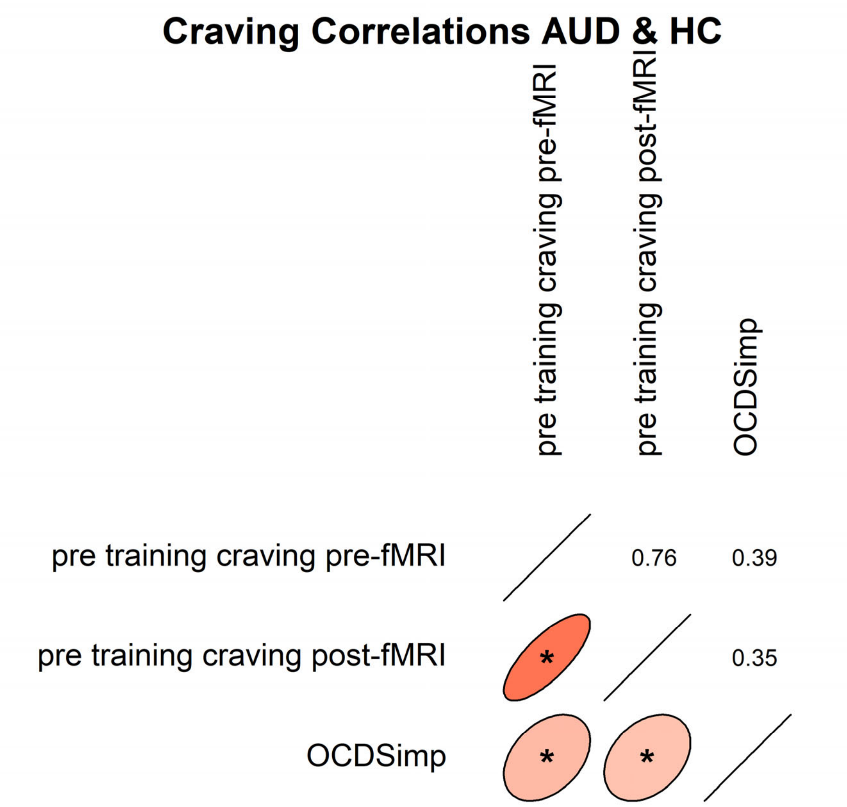


**Supplementary Figure 3.** Correlation matrix showing significant positive correlations between pre- and post-fMRI craving as assessed with a visual analogue scale (VAS) and transsituational craving assessed with the OCDSimp. Deviation extent of ellipse from a circle reflects correlation strength, which is also color-coded. Upper right figure panel displays correlation coefficients (*r*). Asterisks indicate significance $p < 0.05$.

Abbreviations: OCDSimp, Obsessive-Compulsive Drinking scale, imp index; VAS, visual analogue scale (1 – 10) on current craving self-report.

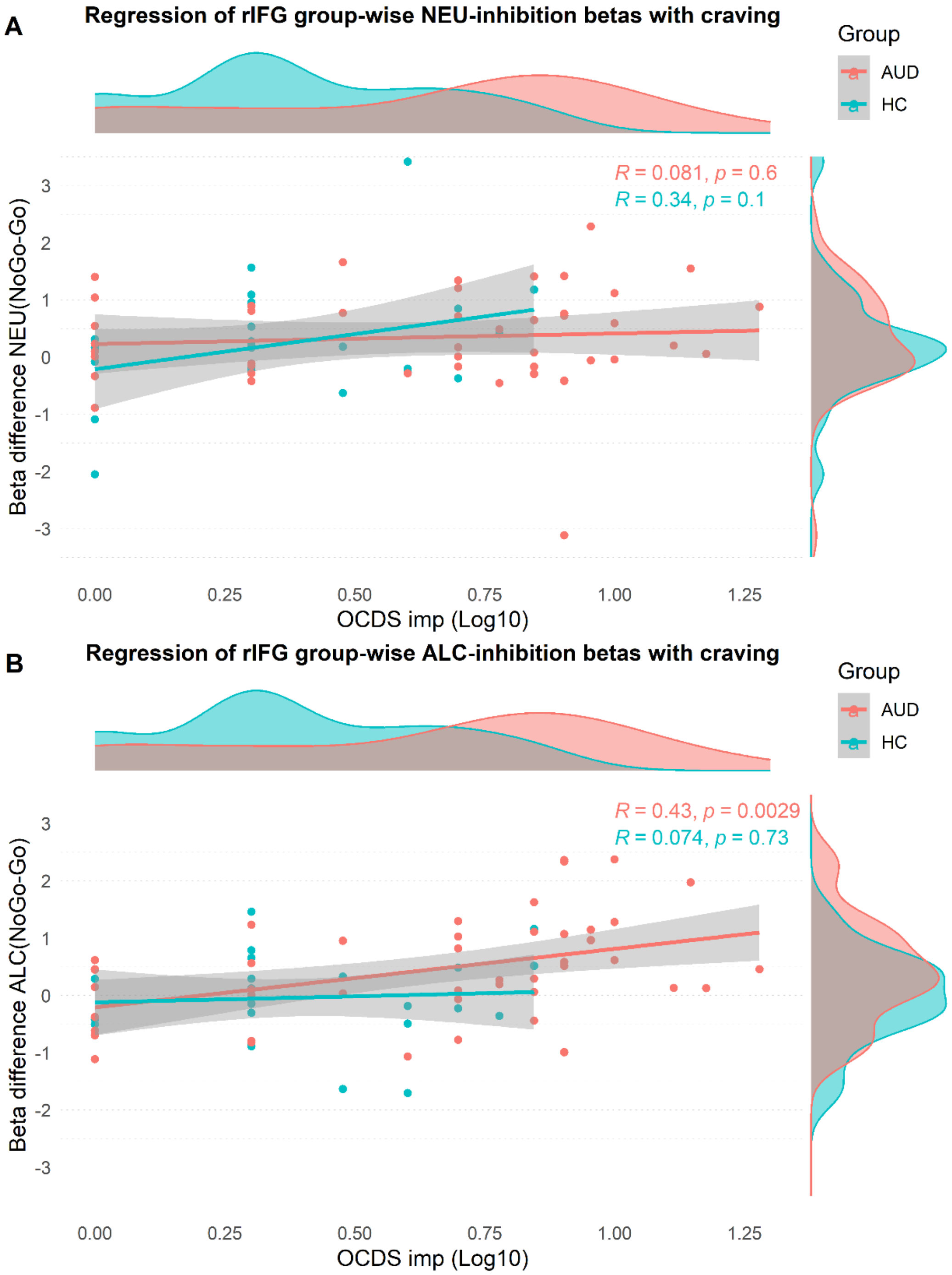


**Supplementary Figure 4.** Visualization of the relationship of rIFG beta-values of alcohol-related inhibition (A) and neutral-related inhibition (B), and log10-transformed OCDSimp scores, stratified for participant group. Disentangling these correlations indicate that activation of the rIFG ROI was specific for alcohol-related inhibition only and was related to craving only in the AUD group.

Abbreviations: AUD: Alcohol use disorder. ALC: alcohol-related stimulus. HC: Healthy controls. NEU: neutral stimulus. OCDSimp: Obesessive compulsive drinking scale – subscale capturing compulsive drinking. rIFG: right inferior frontal gyrus.

References

1. Tschuemperlin, R.M., et al., *Learning to resist the urge: a double-blind, randomized controlled trial investigating alcohol-specific inhibition training in abstinent patients with alcohol use disorder.* Trials, 2019. **20**(1): p. 402.
